# The Khoekhoegowab Personality Inventory: The Comparative Validity of a Locally Derived Measure of Traits

**DOI:** 10.3389/fpsyg.2021.694205

**Published:** 2021-07-20

**Authors:** Amber Gayle Thalmayer, Gerard Saucier, Elizabeth N. Shino, Sylvanus Job

**Affiliations:** ^1^Institute of Psychology, University of Lausanne, Lausanne, Switzerland; ^2^Department of Psychology, University of Oregon, Eugene, OR, United States; ^3^Department of Human Sciences, Psychology Section, University of Namibia, Windhoek, Namibia; ^4^Department of Asian and African Studies, Humboldt University, Berlin, Germany

**Keywords:** Africa, Namibia, majority world, indigenous research, Questionnaire Big Six, comparative validity, psychological disorders, HEXACO

## Abstract

**Objective:** This study explores a personality inventory derived from the results of an indigenous lexical study of personality. From the 272 most commonly used personality descriptors in Khoekhoegowab, the most-spoken of extant *Khoesan* click languages of southern Africa, an 11-factor model of personality-trait structure was identified. Here, the Khoekhoegowab Personality Inventory (KPI) was created based on those results. Its psychometric properties, the convergent and divergent validity of its scales, and its incremental validity over Big Five and Six traits for predicting physical and mental health, religious practice and attitudes, and income are reported.

**Methods:** Two to five key terms were selected for each of 10 KPI scales: Temperance, Prosocial Diligence, Gossip, Honesty/Morality, Temper, Implacability, Humility, Vanity, Resiliency vs. Agitation, and Courage vs. Fear. These 38 total items were administered to a large sample of adult speakers of Khoekhoegowab in Namibia (*N* = 632), together with five imported inventories translated into Khoekhoegowab: the 30-item Questionnaire Big Six (QB6), General Self-Reported Health, the Cascades Mental Health Assessment, the Satisfaction with Life Scale, the Duke Religion Index. The properties and intercorrelations of KPI subscales are explored, and their predictive ability for the other variables is compared to that of the QB6.

**Results:** Due to the small number of items on each scale, poor internal consistency was anticipated, but the KPI scales' properties were somewhat better than those of the QB6. R-square change by the inventories as a whole, after accounting for age and gender, indicted that the KPI scales explained more variance than the QB6 scales in almost all criterion variables. Replication of established associations for Big Six traits was mixed: associations were largely as expected for Resiliency, Conscientiousness, and Honesty, but less so for Agreeableness and Extraversion.

**Conclusions:** The KPI had some advantages over the QB6 in predicting physical and mental health. In particular, the four items of Resiliency vs. Agitation predicted lower scores on all physical and mental problem scales. Given psychological-care needs in Namibia, this might be used as a non-intrusive screener. Measurement challenges common to both surveys are discussed, possible solutions, and the utility of higher-order structures are discussed.

## Introduction

The most common way to measure personality traits around the world now is with Big Five inventories. Inventories based on this model, including dimensions of Extraversion, Agreeableness, Conscientiousness, Emotional Stability, and Openness, have been translated and imported throughout the world, even to hunter-gatherer groups in the Amazon (Gurven et al., 2013). The model, however, was based on lexical studies of personality in the United States, Germany, and the Netherlands (Hofstee et al., 1997), three closely related languages and cultural contexts. The lexical methodology is uniquely well-suited to cross-cultural comparisons that might address the complex question of universality. However, subsequent studies in over a dozen other languages often only tested for the Big Five (as summarized by Thalmayer et al., 2020a, Supplementary Table 1), perhaps to avoid contradicting influential members of the field who insisted on the universality of this model (e.g., McCrae and Costa, 1997). Unsurprisingly, large recent survey studies indicate measurement validity problems for Big Five inventories in the majority world, outside industrialized Western countries (e.g., Ludeke and Larsen, 2017; Laajaj et al., 2019). A six-factor structure (called HEXACO or the Big Six) adding a scale with content related to honesty and integrity vs. taking advantage of others, demonstrated better convergence among a larger group of lexical studies (Ashton et al., 2004; Saucier, 2009), but later evidence suggests this model does not arise everywhere (Thalmayer et al., 2020a,b). The current study explores an alternative approach, creating an “indigenous” personality inventory based on the results of a local lexical study, and comparing it directly to an imported inventory of Big Five and Big Six traits. This builds on prior efforts to assess the significance of local content, for example, the Chinese Personality Inventory, which has been found to provide incremental validity beyond Big Five scales in predicting life, career, and health outcomes in Chinese and Western samples (Cheung et al., 2013).

A recent lexical study of personality explored the most commonly used person-descriptive terms in Khoekhoegowab (Thalmayer et al., 2020a). Khoekhoegowab, literally “the Khoekhoe language,” also referred to as Nama, Damara, or Nama/Damara, is the most widely-spoken of ~15 extant Khoesan (also *Khoisan*) click languages of southern Africa (Haacke, 2011; Güldemann and Fehn, 2014). Two main groups in Namibia, with differing cultural and ethnic backgrounds, speak Khoekhoegowab today. The Damara were hunter-gatherers and later pastoralists related genetically to Bantu speakers (Pickrell et al., 2012; Pakendorf, 2014) who may have lived in the area now known as Namibia before the arrival of Khoisan groups, including the Nama, from other parts of southern Africa (Barnard, 1992). Compared to the Damara, the Nama traditionally had larger clans, more elaborate political organization, and more emphasis on hierarchy and the role of chiefs (Barnard, 1992). Clan memberships and the royal families associated with each are still important in Nama culture. During apartheid, Damara people were restricted to a central and north-western part of the country, and Nama to the southern part of the country around Keetmanshoop and Mariental. While this separation is no longer maintained by law, it is still largely in place culturally. Currently, Khoekhoe-speakers comprise about 11% of the population in Namibia (Namibia Statistics Agency, 2013), making this the second most-commonly spoken “home language” of the 10 languages that are available in schools and at the university level (Frydman, 2011; Namibia Statistics Agency, 2013).

The exploration of personality description in Khoekhoegowab was initially motivated by basic science, rather than practical, questions. The goal was to build a local model of personality, in addition to those built in Maa and in Supyire-Senufo (Thalmayer et al., 2020b), in order to compile evidence from at least one each of the roughly three main language families in Sub-Saharan Africa, among cultural groups with different ethnographic characteristics in far-separated regions. This approach enabled researchers to represent some of Africa's great linguistic and cultural diversity. These three lexical studies used the same methods that led to the Big Five in English, German and Dutch, so that the replication and “universality” of this and other proposed structural models could be directly tested. However, community samples rather than college students, and a more systematic approach to data analysis, comparing data treatments and rotation strategies directly rather than relying on arbitrary traditions, were implemented. In addition to these important tests of replication for the imported models, the local models identified in these three studies provide insight into the particular concerns and interests of the local populations. The ways that these differ from the Big Five shed light on contextual differences. For example, Extraversion, considered a key distinction in the United States, appears to be of much less importance in African languages. This trait is more likely to be talked about in contexts where there is a lot of interaction with strangers and high relational mobility. Instead, in Supyire-Senufo society, horticulturalists living in small villages in Mali, Diligence vs. Laziness emerged as an important local trait, including a cluster of commonly-used words to denote subtle differences in degree (Thalmayer et al., 2020b). This is the something that a lexical study can tell us: What individual differences have people tended to discuss in this particular context? In North America and Northern Europe we have seen that people discuss qualities related to Extraversion, Agreeableness, Conscientiousness, Emotional Stability, and Openness. But even if these traits can be translated and imported into other contexts, the evidence is that these are not the traits that arise naturally outside a small cluster of Western industrialized societies.

In Khoekhoegowab, a systematic process of comparing potential models of the 272 most-commonly-used person-descriptive terms for robustness led to identification of an “optimal emic model” of 11 factors. The first factor, termed Intemperance (renamed Temperance for the KPI) contrasts substance abuse and other externalizing behaviors with being a religious person. The second, Prosocial Diligence, contrasts readiness to help and work, and attentive, orderly, clean conduct with work avoidance, sloppiness, and laziness. It was moderately correlated with marker scales for Big Five and Big Six Conscientiousness and Agreeableness. Gossip contrasts asking too many questions, spreading lies and rumors, and talking others down, with being a good and wise person. It had small negative correlation with Agreeableness and Honesty/Propriety marker scales. Immorality (renamed Honesty/Morality here) contrasts being deceitful and dishonest with being trustworthy; it was moderately negatively correlated with Honesty. Bad Temper (renamed Temper) captures a tendency for reactive aggression and anger. This is related to Implacability, which contrasts being envious, difficult, and dissatisfied with being a helpful, humble person, but seems to capture a quieter, rather than openly hostile, side of disagreeableness. They are both moderately correlated with Big Five Agreeableness, but Temper more specifically with Big Six low Agreeableness, which functions to distinguish reactive from predatory aggression (at the low end of Honesty/Propriety; Thalmayer, 2018). Implacability is also moderately negatively correlated with Conscientiousness, suggesting more passive aggression.

A factor termed Predatory Aggression included criminal, sinister, and violent content. Unsurprisingly, this factor was uncorrelated with any imported scales because such evaluative content has typically been excluded from lexical studies and personality inventories. It was also excluded from the KPI for this reason and because the relevant terms are rather offensive; all were deemed unsuitable for a self-report inventory. The eighth and ninth factors made a rather specific distinction. Haughty Self-Respect (renamed Humility here), contrasts pride, haughtiness, arrogance, and positive aspects of self-respect with peripheral loadings related to religiousness, compassion and humility. This factor suggests some ambivalence among Khoekhoe speakers: an appreciation of the merits of dynamic self-confidence, coupled with awareness that such qualities can conflict with a desire to show humility. It was largely uncorrelated with marker scales, suggesting quite culture-specific content. Vanity/Egotism (renamed Vanity here), including terms for vanity, boastfulness, and pretentiousness, indicates a more clearly negative egocentrism and was moderately negatively correlated with Big Five Conscientiousness and Agreeableness, and Big Six Honesty. Resilient vs. Agitated contrasts having a good and happy character with being restless and anxious, was moderately positively correlated with Agreeableness and Resiliency. Courage vs. Fear contrasts positive dynamic courage with being withdrawn, mistrustful, and timid, and was correlated with Big Six Resiliency and Big Five Emotional Stability.

The current study makes an exploration of the potential practical utility of this structural model for traits that were identified in Khoekhoegowab. The 10-factor, 38-item Khoekhoegowab Personality Inventory (KPI) was created drawing on key terms for these dimensions, and its psychometric properties and convergent and divergent validity are assessed and contrasted with those of the 30-item Questionnaire Big Six translated into Khoekhoegowab. We hypothesized in a preregistered analysis plan that overall, the KPI would more strongly associate with criterion variables for physical and mental health (psychological disorders, well-being, physical health) and with religiosity than the Questionnaire Big Six (QB6). Specific associations were hypothesized for the QB6 based on the prior literature for associations with Big Five and Big Six traits from other cultural contexts, and for KPI based on face validity, detailed below.

## Materials and Methods

### Participants

Participants were 645 adult native speakers of Khoekhoegowab in Namibia. They were recruited from throughout the country, including central, eastern, northern and southern Namibia. Demographic information collected included age, gender, home language, participant and parents' level of schooling, household income, employment level, and location of survey-interview. Details are provided in Table 1.

**Table 1:** Sample Characteristics.

	**Participant**		**Female-Caregiver**		**Male-Caregiver**	
	***n***	**(%)**	***n***	**(%)**	***n***	**(%)**
**Gender**						
Male	303	(47.0)				
Female	342	(53.0)				
**Employment status**						
Not currently working	236	(36.6)				
Students	139	(21.6)				
Work at home or other unpaid work	67	(10.4)				
Seeking paid work	120	(18.6)				
Occasional paid work	96	(14.9)				
Regular part-time paid work	51	(7.9)				
Regular full-time paid work	226	(35.0)				
**Monthly Income in Namibian Dollars**						
None	98	(15.3)				
Between N$1 and 500	165	(25.7)				
Between N$500 and 1,500	105	(16.4)				
Between N$1,500 and 3,000	86	(13.4)				
Between N$3,000 and 5,000	56	(8.7)				
Between N$5,000 and 10,000	73	(11.4)				
Over N$10,000	59	(9.2)				
Not reported	3	(0.5)				
**Level of Education**						
Did not finish primary	28	(4.4)	222	(35.9)	193	(31.7)
Grade 7 primary	67	(10.4)	94	(15.2)	92	(15.1)
Grade 10 secondary	213	(33.1)	139	(22.5)	117	(19.2)
Grade 12 secondary	178	(27.7)	113	(18.3)	117	(19.2)
Vocational after grade 10 or 12	30	(4.7)	18	(2.9)	29	(4.8)
University or diploma	86	(13.4)	12	(1.9)	22	(3.6)
University Bachelor's degree	35	(5.4)	13	(2.1)	20	(3.3)
Masters/post-graduate degree	6	(0.9)	8	(1.3)	18	(3.0)
Not reported	2	(0.3)	26	(4.0)	37	(5.7)

*N = 645, aged 18–62 years (M = 34.8; SD = 11.1)*.

### Materials

Aside from the Khoekhoegowab Personality Inventory, the other surveys used in this study were originally created in English. These surveys are the Questionnaire Big Six (QB6), the Duke University Religion Index (DUREL), the Satisfaction with Life Scale (SWLS), the Cascades Mental Health Assessment (CMHA), and General Self-Reported Health (GSRH). They were translated into Khoekhoegowab for this project, involving multiple professional translators, linguists, and native speaker psychologists, following a process using expert panels as defined by the World Health Organization (https://www.who.int/substance_abuse/research_tools/translation/en/).

#### Khoekhoegowab Personality Inventory (KPI)

To develop this inventory for the current project, 38 terms were chosen from among the 272 administered in the 2018 lexical study. Two to five were chosen for each of 10 factors of the optimal emic model identified in that project (described above). Item selection was from among terms with a loading of 0.30 or higher on the relevant factor; the number of possible items for each factor thus ranged from 7 to 21, with an average of 13.7. Choices within the pool for each factor emphasized the highest loadings on the dimension, and univocal terms (those with cross loadings never above 50% of the main loading), with consideration for the balance of forward and reverse-keyed items—scales were either unipolar (all items loading the same direction) or they included an equal number of forward and reverse-keyed items. We avoided selecting two terms with the same root and we sought coverage of each dimension's content, seeking to incorporate all key aspects.

An instruction was given, a translation version of: “I will read you statements people can use to describe themselves. Each time say how true this is for you.” Items were framed into statements using three possible stems depending on the word type: adjective (I am …); verb (I like to …); or noun for a quality (I have …). Items were answered on the same six-point scale used for the QB6, in terms of how true the item is for describing oneself: very untrue, moderately untrue, slightly untrue, slightly true, moderately true, very true.

Given the lack of prior research using this measure, analyses were largely exploratory. Hypotheses were developed based on face validity leading to the following expectations for significant associations:

- Temperance: negative with Substance Abuse and being male, positive with DUREL scales and possibly GSRH.
- Prosocial Diligence: positive with Work Engagement, SWLS, possibly GSRH.
- Gossip: positive with Conflict scales.
- Honesty/Morality: positive with DUREL scales, and SWLS.
- Temper: positive with Anger and GSRH.
- Implacability: positive with Anger, negative with DUREL scales and SWLS.
- Humility and Vanity: positive with both Conflict scales.
- Resiliency vs. Agitation and Courage vs. Fear: both positive with CMHA Total, Depression and Anxiety; negative with SWLS and GSRH.

#### Questionnaire Big Six (QB6)

The 30-item cross-cultural QB6 (Thalmayer and Saucier, 2014) is an inventory assessing six broad personality traits (Conscientiousness, Agreeableness, Honesty/Propriety, Resiliency, Extraversion, Originality/Openness) with five items each. The items were chosen based on evidence of their cross-cultural applicability in a study comparing responses from 26 countries and languages. The instruction before the items were read and the response options were the same as for the KPI.

Prior research with Big Five/Six traits in other cultural contexts provided hypotheses to test for replication in the current study. These are listed here and are graphically displayed in the tables below. In terms of gender, women have been seen to score higher on Extraversion, Agreeableness, Neuroticism (Lippa, 2010). In terms of age, cross-sectional studies of Big Five traits show older people to score higher on Agreeableness, Conscientiousness, and Emotional Stability (Soto et al., 2011). Income and work success have been seen to be higher in those with higher Conscientiousness and Emotional Stability (Ozer and Benet-Martínez, 2006). A meta-analysis of association between religiousness and Big Five traits from 19 countries reported consistent small positive correlations with Agreeableness, Conscientiousness, and Extraversion (Saroglou, 2010); studies including HEXACO Honesty found it to be positively associated with religiousness generally and with DUREL Intrinsic Religiosity specifically (Aghababaei et al., 2014). The Satisfaction with Life Scale has been shown to positively associate with Emotional Stability and Extraversion (Schimmack et al., 2004). Self-reported health has been associated positively with Conscientiousness and negatively with Neuroticism (Kööts–Ausmees et al., 2016). Associations with common psychological problems reported in large meta-analyses (Malouff et al., 2005; Kotov et al., 2010) include mood disorders with lower Emotional Stability and Extraversion, and substance abuse with lower Conscientiousness and higher Extraversion. Extraversion has been associated with attention problems (Nigg et al., 2002), and low Honesty with substance abuse (Saucier, 2009), aggression toward romantic partners (Mogilski et al., 2019), and violent behavior (Pailing et al., 2014).

#### The Duke University Religion Index (DUREL)

This 5-item measure of religious engagement was designed to assess religiosity in regards to health and epidemiological outcomes (Koenig and Büssing, 2010). Its three subscales distinguish between three aspects of religiosity: Organized Religious Activity, Non-Organized Religious Activity, and Intrinsic Religiosity. The index has been shown to be a valid and reliable in diverse contexts (Lucchetti et al., 2012; Chen et al., 2014; Hafizi et al., 2014). Responses are on a six-point Likert scale with slightly different terminology linked to numerical values for each question. The total score had good reliability in this sample, α = 0.82.

#### The Satisfaction With Life Scale (SWLS)

This 5-item measure assesses well-being in terms of global cognitive judgments of satisfaction with one's life (Diener et al., 1985). The scale had acceptable reliability in this sample, α = 0.74.

#### General Self-Reported Health (GSRH)

Physical health was rated with one item “In general, would you say your health is...?” on a 5-point scale from poor to excellent. A meta-analysis of 22 studies has shown this one-item self-assessment to correlate highly with longer or more invasive measures of health status and to be strongly associated with risk of death over 5 years (DeSalvo et al., 2006).

#### Cascades of Mental Health Assessment (CMHA)

The Cascades Mental Health Assessment [CMHA; Thalmayer et al. (in preparation)] is a 59-item, 9-subscale measure of common psychological problems designed for screening in a normal adult population. Items are specific and behavioral, and the response scale assesses concrete frequency in terms of days out of the last month. These qualities are intended to help avert reference group effects to maximize validity in particular when comparing across groups (linguistic, national, gender, education level, etc.). The items can be combined into a total score or divided into 10 subscales: Substance Abuse, Anxiety, Depression, Post-Traumatic Stress, Stress, Sleep Issues, Anger, Work Disengagement, Interpersonal Conflict, and Partner Conflict. For this study, additional items to measure attention problems (5 items) and psychosis (2 items) were adapted from other inventories and included for a total of 64 items administered.

### Procedure

This study was part of a larger data collection effort in three languages conducted in Namibia in 2019. The full project administered surveys also to speakers of English and of Oshiwambo, but the KPI was only administered to Khoekhoegowab-speakers and only this sample is reported on here. Ethical review of the study plan was made by University of Namibia and a research permit was issued by Namibia's National Commission on Research Science and Technology. A team of 15 interviewers recruited participants and collected 20–100 surveys each. Interviewers were graduates of the psychology programs of the University of Namibia and primary- or secondary-level schoolteachers of the Khoekhoe language. Almost all interviewers had previously collected data with the same research team. Data collection occurred in the 8-week period following a training weekend. Written informed consent was obtained from all participants. The surveys were conducted as private interviews of ~40 min each, with the interviewer reading the questions to participants and referring to response options both verbally and in a written form, on a sheet shown to participants. This is because while Khoekhoe is the mother tongue spoken by participants and commonly used in social and business contexts in the areas where interviews occurred, schooling is often in English (Afrikaans until 1990), and thus many participants are not highly literate in their mother tongue. Interviewers noted on surveys their own name, the area in which the interview occurred, and the gender they perceived the participant to be. They asked participants to report their home language, age, employment status, and household income and their own and their parents' (or caregivers') level of schooling. No other identifying information was recorded.

### Analyses

#### Data Exclusions

Based on criteria described in the pre-registered analysis plan, 37 of 682 total cases were excluded from analysis. This was due to: being marked for exclusion by the interviewer, either because it was not completed or because the interviewer felt that it was not reliably completed (*n* = 2); participant under age 18 (*n* = 2); more than 15% of responses missing (*n* = 7); all CMHA items given same response (*n* = 13); standard deviation on QB6 <0.50, indicating virtually no variation in responses (*n* = 2); extreme outlier on person-to-total correlations for QB6 and CMHA, indicating likely random responding (*n* = 11). This left an analytic sample of 645 cases.

#### Scale Exploration

The properties of the KPI and the QB6 were assessed and explored using standard psychometric indices, parallel analysis (O'Connor, 2000), and principal components analysis (PCA). The fit of each inventory to its intended structure was additionally assessed using confirmatory factor analysis (CFA).

#### Associations and Comparative Validity

The zero-order Pearson correlations among the personality scales and between personality scales and the other measures are reported. In multiple regression analyses for each criterion, age and gender were entered at step one and the set of scales for the KPI and separately for the QB6 were entered at step two. Prediction was at the level of scores on each inventory in part to minimize Type I error. Results are reported in terms of an overall change in *R*^2^ after accounting for age and gender. Scales within each inventory with a coefficient significant at *p* < 0.01 or 0.05 are noted. Additionally, incremental validity for the KPI over the QB6 was directly tested by entering age, gender, and all QB6 scales at step one, and KPI scales at step two.

## Results

### Scale Properties and Exploration

Psychometric properties of the KPI and QB6 are reported in Table 2. Due in part to the small number of items on each scale, poor internal consistency was anticipated. In fact, the KPI scales' alpha values for eight of the 10 scales (0.34 to 0.66) were generally better than those of the slightly longer QB6 scales (0.26 to 0.53). Two KPI scales had quite low alpha values: Resiliency vs. Agitation (0.27) and Courage vs. Fear (0.03). Resiliency vs. Agitation included two moderately-correlated pairs of items: two forward-keyed items, referring to being tolerant and of a happy disposition, and two reverse-keyed items, about being restless, fidgety and anxious. These pairs were virtually uncorrelated with each other. Courage vs. Fear had the same structure, with two items referring to being adept and brave, and two to being jumpy and standoffish. In this case, however, all correlations were quite low (0.00–0.14).

**Table 2:** Psychometric Properties of the Khoekhoegowab Personality Inventory (KPI) and Questionnaire Big Six (QB6) in Translation to Khoekhoegowab.

	***N***	**items**	***M***	***SD***	**α**	**α ^**standardized**^**	**v.i. *r***
**Khoekhoegowab Personality Inventory**							
Temperance	640	5	2.35	1.00	0.634	0.631	0.024
Prosocial Diligence	639	4	4.87	0.96	0.595	0.602	0.007
Gossip	642	2	2.10	1.20	0.644	0.644	-
Honesty/Morality	640	4	2.23	1.04	0.652	0.657	0.005
Temper	639	4	3.08	1.22	0.664	0.665	0.007
Implacability	638	5	2.31	0.83	0.465	0.471	0.007
Humility	642	3	3.50	1.10	0.344	0.340	0.007
Vanity	641	3	3.12	1.26	0.572	0.572	0.000
Resiliency vs. Agitation	640	4	4.21	0.85	0.285	0.273	0.044
Courage vs. Fear	639	4	3.66	0.86	0.031	0.034	0.010
**Questionnaire Big Six**							
Agreeableness	632	5	3.37	0.87	0.371	0.370	0.010
Extraversion	633	5	3.85	0.82	0.255	0.253	0.008
Originality	629	5	4.13	0.77	0.270	0.292	0.012
Resiliency	641	5	3.38	0.84	0.335	0.326	0.020
Conscientiousness	634	5	4.25	0.94	0.533	0.532	0.003
Honesty	637	5	4.34	0.97	0.497	0.501	0.008

*v.i.r, variance of inter-item correlations, where lower values indicate better unidimensionality (Clark and Watson, 1995)*.

Although 10 factors were expected for the 38 items of the KPI and six for the 30 items of the QB6, in both cases parallel analysis suggested that only five factors in raw data and eight factors in ipsatized data were larger than would be expected by chance in a dataset of this size. Inspection of the rotated factors in PCA for models of the intended and suggested sizes indicated that many respondents appeared to have had a tendency to “perseverate” on a specific response. The items for both inventories were presented in a set order from a printed page. In both cases, items from different subscales were separated, such that, for example, Agreeableness items on the QB6 appeared next to Extraversion and Resiliency items, but never next to each other. However, PCA results revealed a tendency for items with subsequent numbers to appear on the same components. This suggests that some participants may have tended to repeat an answer, perhaps because the items were hard to relate to themselves or out of a lack of an opinion on what answer to give. After calculating an indicator of perseveration (by summing the squared differences between every pair of adjacent items, for an overall indicator in which lower scores indicate a higher degree of perseveration across items), *post hoc* exploration suggested that removing the 20% of cases with the most extreme scores reduced this pattern to some extent. However, subsequent analyses reported here used the original intended data.

CFA fit for the QB6 in Khoekhoegowab, χ^2^ (390) = 2109.01, RMSEA 0.086 (CI: 0.083, 0.090), SRMR = 0.085 CFI = 0.688, TLI = 0.652, was slightly poorer than that of the KPI χ^2^ (620) = 3,550, RMSEA 0.088 (CI: 0.085, 0.091), SRMR = 0.095 CFI = 0.764, TLI = 0.733. Neither met standard benchmarks for good fit (e.g., Hu and Bentler, 1999) but they compare favorably to those typically seen for multi-dimensional personality inventories, even in the language of their original development (Hopwood and Donnellan, 2010) and to the QB6 in other translations (Thalmayer and Saucier, 2014).

The correlations and intercorrelations of the KPI and the QB6 subscales are reported in Table 3. KPI scales were in some cases moderately associated with each other, with intercorrelations of <0.10 to just over 0.50 in magnitude. These patterns of correlation suggest “clusters.” Five subscales, namely Temperance, Prosocial Diligence, Gossip, Honesty/Morality, and Implacability inter-correlate with magnitudes of about 0.33 to 0.55 with each other. Humility and Vanity form a moderately correlated pair (*r* = 0.51). To a lesser extent, Resiliency vs. Agitation belongs to the first group, and Temper associates with both groups.

**Table 3:** Khoekhoegowab Personality Inventory (KPI) and Questionnaire Big Six (QB6) Scale Correlations and Intercorrelations.

	**Khoekhoegowab Personality Inventory**										**QB6**				
**KPI**	**1**	**2**	**3**	**4**	**5**	**6**	**7**	**8**	**9**	**10**	**A**	**E**	**O**	**R**	**C**
1 Temperance															
2 Prosocial Diligence	**0.50**														
3 Gossip	**−0.38**	**−0.41**													
4 Honesty/Morality	**−0.51**	**−0.55**	**0.54**												
5 Temper	**−0.33**	−0.26	0.28	**−0.48**											
6 Implacability	**−0.48**	**−0.54**	**0.37**	**−0.48**	**0.38**										
7 Humility	−0.16	−0.05	0.16	−0.24	**0.34**	0.15									
8 Vanity	−0.19	−0.12	0.27	−0.30	**0.33**	0.26	**0.51**								
9 Resiliency vs. Agitation	**0.32**	**0.31**	−0.24	**0.34**	**−0.34**	**−0.41**	−0.03	−0.13							
10 Courage vs. Fear	0.09	0.15	−0.07	0.11	0.00	−0.07	0.05	0.10	0.21						
QB6															
Agreeableness	0.15	0.12	−0.07	0.17	**−0.48**	−0.20	−0.17	−0.11	0.18	−0.01					
Extraversion	0.10	0.21	−0.05	0.11	−0.04	−0.15	0.04	0.04	0.12	0.15	0.03				
Originality	0.28	**0.32**	−0.19	0.22	−0.12	−0.26	0.07	0.07	0.28	0.14	0.15	0.17			
Resiliency	0.01	−0.09	0.02	0.06	−0.17	−0.05	−0.07	−0.07	0.30	0.20	0.17	0.01	0.09		
Conscientiousness	**0.44**	**0.50**	**−0.32**	**0.39**	−0.15	**−0.39**	−0.05	−0.13	0.27	0.15	0.06	0.09	**0.32**	0.08	
Honesty	**0.42**	**0.46**	**−0.33**	**0.46**	−0.25	**−0.39**	−0.14	−0.21	0.28	0.09	0.12	0.13	0.29	0.07	**0.43**

*Moderate correlations ≥0.30 in magnitude are bolded for emphasis*.

Moderate correlations of 0.30 to 0.50 between KPI and QB6 scales suggest similar patterns to those reported by Thalmayer et al. (2020a, Table 3), in that case between the full factor scores from the lexical PCA analyses, with marker scales for the Big Five and Six drawn from Khoekhoegowab terms. In the current data, Temperance is again moderately correlated with Conscientiousness, here also with Honesty but not with Extraversion. Prosocial Diligence is again moderately correlated with Conscientiousness, but less so here with Agreeableness; Gossip again with Honesty, now also Conscientiousness; Honesty/Morality again with Honesty, now also Conscientiousness but not Agreeableness; Temper again with Agreeableness but not Extraversion; Implacability again with Conscientiousness, now also Honesty, but not Agreeableness. The four remaining scales had no moderate correlations with Big Six scales, although in the prior data Resiliency vs. Agitation and Courage vs. Fear correlated with Resiliency. Moderate correlations were also seen in the prior study for Vanity with Big Five (but not Big Six) Conscientiousness, and for both Vanity and Resiliency vs. Agitation with Big Five (but not Big Six) Agreeableness. Humility was not correlated with outside traits in either study.

### Associations and Comparative Validity

**Figure 1:**
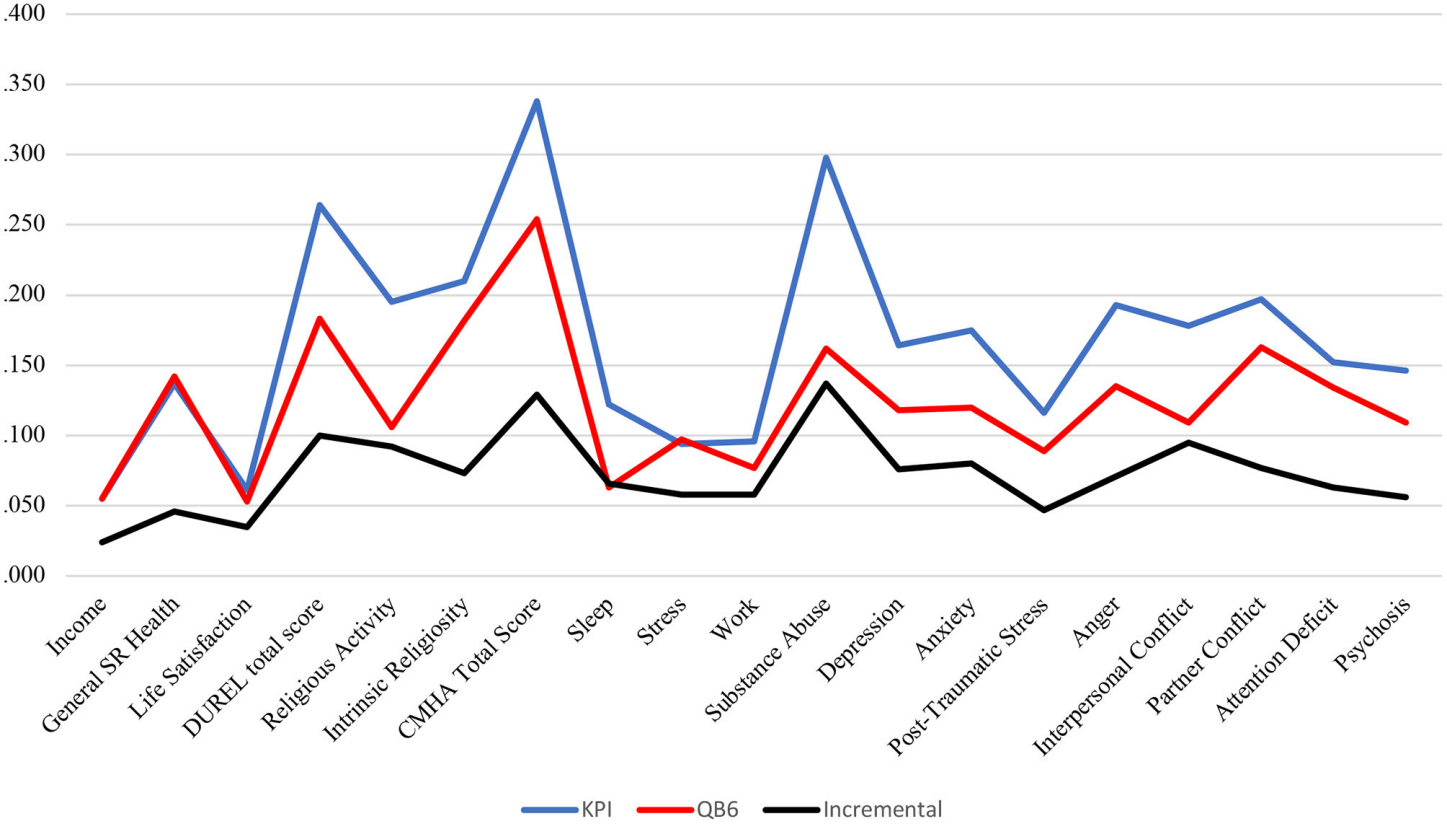
Comparative Change in *R*^2^ for KPI and QB6 in Predicting Criterion Variables, Accounting for Age and Gender. Duke Religion Index (DUREL); Cascades Mental Health Assessment (CMHA). Incremental = age, Gender, and all QB6 Scales Entered at Step one, KPI Scales at Step two.

Zero-order Pearson correlations between the KPI and QB6 scales and demographic and criterion variables are reported in Table 4. *R*^2^ change for predicting criterion variables with the personality inventories after accounting for age and gender are reported in Table 5. For all outcome variables with both personality inventories, the change in *R*^2^ was always significant at *p* < 0.001. Together, the KPI scales predicted from a low of 6% (income) to a high of 34% (CMHA total score) of the variance in criterion variables. The range for the QB6 scales as a whole was slightly lower, from 5% (life satisfaction) to 25% (CMHA Total Score). The change in *R*^2^ values for the two inventories for the 18 criterion variables/scales is displayed graphically in Figure 1. For almost all criteria, the KPI scales explained more variance than the QB6 scales. Direct tests of incremental validity for the KPI over the QB6, also shown in Table 5 and Figure 1, indicate that this was significant for all outcome criteria except for income. The KPI predicted from a low of 4% (life satisfaction) to a high of 14% (Substance Abuse) over and above the QB6 scales, age, and gender.

**Table 4:** Correlations Between Personality Scales and Demographic and Criterion Variables.

	**Khoekhoegowab Personality Inventory**										**Questionnaire Big Six**					
	**1**	**2**	**3**	**4**	**5**	**6**	**7**	**8**	**9**	**10**	**A**	**E**	**O**	**R**	**C**	**H**
Age	0.10	0.03	0.03	0.10	−0.12	−0.06	−0.21	−0.11	0.03	0.13	0.06	0.05	−0.04	0.13	0.05	0.08
Gender	−0.17	−0.13	−0.08	−0.12	0.02	0.06	0.11	0.02	0.00	0.01	−0.05	−0.06	−0.05	0.16	−0.08	−0.14
Income	0.17	0.08	−0.03	0.13	−0.06	−0.13	−0.05	0.00	0.17	0.13	0.03	0.02	0.15	0.11	0.18	0.15
Education	0.14	0.05	−0.07	0.13	−0.12	−0.07	−0.01	0.01	0.14	0.04	0.03	−0.02	0.28	0.12	0.18	0.13
General SR Health	0.18	0.24	−0.22	0.17	−0.07	−0.21	0.08	0.03	0.25	0.17	0.00	0.04	0.23	0.14	0.29	0.20
Life Satisfaction	0.17	0.08	−0.05	0.11	−0.06	−0.10	0.07	0.05	0.13	0.14	0.03	0.02	0.13	0.11	0.18	0.13
DUREL Total Score	**0.48**	**0.38**	−0.22	**0.36**	−0.18	**−0.35**	−0.06	−0.12	0.27	0.18	0.13	0.12	0.21	−0.02	**0.38**	**0.37**
Religious Activity	**0.44**	0.29	−0.17	0.28	−0.16	−0.24	−0.04	−0.08	0.22	0.16	0.13	0.06	0.20	0.03	**0.31**	0.26
Intrinsic Religiosity	**0.38**	**0.37**	−0.20	**0.34**	−0.14	**−0.36**	−0.06	−0.12	0.24	0.15	0.09	0.14	0.16	−0.06	**0.33**	**0.38**
CMHA Total Score	**−0.38**	**−0.33**	**0.33**	**−0.42**	0.29	**0.37**	0.15	0.17	−0.46	−0.15	−0.05	−0.07	−0.20	−0.23	**−0.42**	**−0.38**
Sleep	−0.11	−0.02	0.09	−0.14	0.19	0.11	0.08	0.07	−0.29	−0.12	−0.07	−0.03	−0.06	−0.25	−0.11	−0.10
Stress	−0.03	−0.02	0.09	−0.07	0.11	0.11	0.07	0.04	−0.25	−0.10	0.07	−0.03	−0.02	−0.27	−0.06	−0.09
Work Disengagement	−0.12	−0.10	0.12	−0.19	0.17	0.14	0.09	0.06	−0.23	−0.08	0.03	−0.05	−0.05	−0.16	−0.21	−0.13
Substance Abuse	**−0.53**	**−0.35**	0.28	**−0.38**	0.20	**0.31**	0.11	0.14	−0.28	−0.08	−0.06	−0.07	−0.21	0.00	**−0.33**	**−0.37**
Depression	−0.19	−0.21	0.18	−0.25	0.17	0.25	0.09	0.08	**−0.37**	−0.11	−0.04	−0.10	−0.17	−0.22	−0.24	−0.22
Anxiety	−0.21	−0.18	0.17	−0.26	0.22	0.23	0.12	0.14	**−0.37**	−0.16	−0.06	−0.08	−0.09	−0.25	−0.20	−0.25
Post-Traumatic Stress	−0.16	−0.16	0.13	−0.23	0.21	0.17	0.15	0.16	−0.28	−0.14	−0.06	−0.02	−0.08	−0.23	−0.16	−0.21
Anger	**−0.31**	**−0.33**	0.26	**−0.37**	0.27	**0.33**	0.08	0.11	−0.27	−0.12	−0.07	−0.12	−0.13	−0.03	−0.29	**−0.31**
Interpersonal Conflict	−0.26	−0.22	0.27	**−0.32**	0.24	0.28	0.14	0.09	**−0.31**	−0.08	−0.03	−0.09	−0.14	−0.14	−0.25	−0.25
Partner Conflict	−0.24	−0.26	0.27	**−0.34**	0.19	**0.34**	0.01	0.12	−0.28	−0.12	−0.04	−0.09	−0.16	−0.07	**−0.33**	**−0.34**
Attention Deficit	−0.28	−0.21	0.20	−0.28	0.26	0.23	0.16	0.15	−0.29	−0.12	−0.10	0.00	−0.17	−0.21	−0.29	−0.23
Psychosis	−0.25	−0.24	0.24	**−0.33**	0.18	0.26	0.12	0.11	−0.25	−0.14	−0.02	−0.07	−0.11	−0.08	−0.25	−0.28

*Khoekhoegowab scales: 1 Temperance; 2 Prosocial Diligence; 3 Gossip; 4 Honesty/Morality; 5 Temper; 6 Implacability; 7 Humility; 8 Vanity; 9 Resiliency vs. Agitation; 10 Courage vs. Fear. QB6 Scales: A, Agreeableness; E, Extraversion; O, Originality; R, Resiliency; C, Conscientiousness; H, Honesty. Moderate correlations ≥0.30 in magnitude are bolded for emphasis. Hypothesized associations are underlined*.

**Table 5:** R-Square Change for Criterion Variables After Age, Gender for Inventories as a Whole, Indicating Significant Coefficients by Scale.

				**Khoekhoegowab Personality Inventory**													**Questionnaire Big Six**						
	**KPI**	**QB6**	**Incr**.	**Age**	**Male**	**1**	**2**	**3**	**4**	**5**	**6**	**7**	**8**	**9**	**10**	**Age**	**Male**	**A**	**E**	**O**	**R**	**C**	**H**
Income	**0.055**	**0.055**	0.024	++	+	–								+		++				++		+	
General SR Health	**0.137**	**0.142**	**0.046**	––	++		+							++	++	––	++			++	++	++	++
Life Satisfaction	**0.061**	**0.053**	*0.035*		–	++									+						+	++	
DUREL Total Score	**0.264**	**0.183**	**0.100**			++					–			+	+	++	–	+				++	++
Religious Activity	**0.195**	**0.106**	**0.092**		–	++									+	+	–	+				++	+
Intrinsic Religiosity	**0.210**	**0.182**	**0.073**			++	+				––				+	+			+		–	++	++
CMHA Total Score	**0.338**	**0.254**	**0.129**		–	––			–			+		––			–				––	––	––
Sleep	**0.122**	**0.063**	**0.066**				+			+				––							––		
Stress	**0.094**	**0.097**	**0.058**									+		––				++			––		–
Work Disengagement	**0.096**	**0.077**	**0.058**						–					––							––	––	
Substance Abuse	**0.298**	**0.162**	**0.137**		+	––			–					––			+					––	––
Depression	**0.164**	**0.118**	**0.076**											––							––	––	––
Anxiety	**0.175**	**0.120**	**0.080**											––							––	–	––
Post-Traumatic Stress	**0.116**	**0.089**	*0.047*	–										––		––					––		––
Anger	**0.193**	**0.135**	**0.071**						–					–								––	––
Interpersonal Conflict	**0.178**	**0.109**	**0.095**	–	–			+	–			+	–	––		–	–				–	––	––
Partner Conflict	**0.197**	**0.163**	**0.077**		–				––		++			–								––	––
Attention Deficit	**0.152**	**0.134**	**0.063**	–		–								––		––					––	––	–
Psychosis	**0.146**	**0.109**	**0.056**						––					––		–						––	––

*Khoekhoegowab scales: 1 Temperance; 2 Prosocial Diligence; 3 Gossip; 4 Honesty/Morality; 5 Temper; 6 Implacability; 7 Humility; 8 Vanity; 9 Resiliency vs. Agitation; 10 Courage vs. Fear. QB6 Scales: A, Agreeableness; E, Extraversion; O, Originality; R, Resiliency; C, Conscientiousness; H, Honesty. Bolding for change in R^2^ indicates it was significant at p < 0.01, italics indicate that it was significant at p < 0.05. Incr. = age, gender, and all QB6 scales entered at step one, KPI scales at step two. Hypothesized associations are shaded in gray. For coefficients, ++ / –– means it was significant at p < 0.01; +/– = coefficient suggestive at p < 0.05*.

In Tables 4, 5, the hypotheses detailed above in methods are indicated along with the observed associations. In Table 5, the significant scale coefficients are shown graphically. There it can be seen that in total, 35 “face valid” predictions were made for specific KPI scales. Of these, 16 were supported, and 19 were not. Temperance was indeed positively associated with the DUREL scales and negatively with Substance Abuse, but not with GSRH. Additionally, Temperance was positively associated with life satisfaction and negatively associated with income, CMHA total, and Sleep. Prosocial Diligence was indeed positively associated with GSRH, but not with SWLS or Work Disengagement, and it also associated positively with Intrinsic Religiosity and Sleep. Gossip was indeed correlated with Interpersonal, but not with Partner Conflict. Honesty/Morality was not associated with the DUREL scales or SWLS, but instead it predicted fewer problems on the CMHA: total score, Work Disengagement, Substance Abuse, Anger, both conflict scales, and Psychosis. Temper was not associated with Anger or GSRH, but instead with poorer Sleep. Implacability was indeed negatively associated with two of the three DUREL scales, but not with life satisfaction or Anger, and instead predicted more Partner Conflict. Humility did predict more Interpersonal but not more Partner Conflict, and additionally CMHA total and Stress. Vanity predicted less rather than more Interpersonal Conflict, and did not associate with Partner Conflict. Resiliency vs. Agitation had more significant coefficients for criterion variables than any other KPI scale, suggesting its relevance to mental health. As predicted, scores on this scale associated with better self-reported health and lower CMHA total, Depression, and Anxiety scores. They did not associate significantly with life satisfaction, but instead associated with higher income and with lower scores on every CMHA scale. Courage vs. Fear was indeed positively associated with self-reported health and life satisfaction, but not with any CMHA scales; instead, with all DUREL scales.

A total of 35 predictions were also made for specific QB6 scales based on associations established in prior literature. The six of these related to age and gender are only visible in Table 4; all others are interpreted based on the regression results in Table 5. Of these predictions, 21 were supported and 14 were not. Women in Namibia did not score higher on Extraversion, Agreeableness, or Neuroticism, and older people did not score higher on Agreeableness, Conscientiousness, or Emotional Stability. Income, however, was indeed higher for those higher in Conscientiousness, though not for Resiliency, and it was also higher for those higher in Originality/Openness. Self-reported health was associated as expected with Conscientiousness and Resiliency, and it was also associated with Originality/Openness and Honesty/Propriety. Life satisfaction was positively associated with Resiliency, as expected, but not with Extraversion and instead with Conscientiousness. For religiousness, hypotheses were largely met—there were positive associations for the DUREL total score (also for Religious Activity, though specific hypotheses had not been made for that scale) with Agreeableness, Conscientiousness, and Honesty, though not with Extraversion, though Extraversion was associated with the Intrinsic Religiosity subscale. The specific association between DUREL Intrinsic Religiosity with Honesty was also replicated; this subscale was additionally positively associated with Conscientiousness, and negatively with Resiliency.

For psychological problems, the six expected negative associations with Resiliency were found (CMHA total, Sleep, Stress, Depression, Anxiety, and Post-Traumatic Stress); not surprisingly, associations with Resiliency were also significant for Work Engagement, Interpersonal Conflict and attention problems. Likewise both Conscientiousness and Honesty associated negative as expected with three (Work, Substance Abuse, Attention) and four (Substance Abuse, Anger, Interpersonal and Partner Conflict) subscales, respectively, in addition to seven others each: Lower CMHA total score, Depression, Anxiety, and Psychosis for both, additionally the anger and conflict scales for Conscientiousness and Stress, Post-Traumatic Stress, and Attention for Honesty. Agreeableness did not associate negatively with Conflict and Anger, but instead positively with Stress. Extraversion did not associate with Substance Abuse, nor with any other disorder scale. Openness, as expected, was not associated with any psychological disorder scales.

## Discussion

This study describes the development and exploration of an indigenous personality inventory tailored to a specific context, Khoekhoegowab-speakers in Namibia. The 10-factor, 38-item Khoekhoegowab Personality Inventory (KPI) was derived from the results of an indigenous lexical study, and thus theoretically should capture domains of more relevance to the local society. The KPI was compared to Big Five and Big Six traits in terms of their explanatory power for physical health, mental health, well-being and religious engagement, and in terms of demographic items including income. It was hypothesized that the KPI would have better measurement properties in this local context, as well as better predictive validity, similar to the incremental validity seen for the Chinese Personality Inventory, beyond imported scales in predicting important life outcomes in both Chinese and in Western samples (Cheung et al., 2013).

The KPI scales' internal consistency was generally slightly better than that of the QB6 scales, supporting this hypothesis. However, two KPI scales, Resiliency vs. Agitation and Courage vs. Fear, had very low internal consistency. As noted above, each of these scales had two separate content components, which were virtually uncorrelated with each other. Low interitem correlations are not necessarily disqualifying for a short unidimensional scale, and do not harm predictive ability. As a case in point, Resiliency vs. Agitation had more significant associations with outcome criteria (16 total) than any other KPI scale, suggesting that these items are strongly predictive of the physical and mental health outcomes of interest in this study. Courage vs. Fear also had five significant coefficients with regard to outcome criteria: with self-reported health, life satisfaction, and the three DUREL scales. These were not the associations anticipated for this scale, however. Future work should better explore the local meaning and usage of the terms on the Courage vs. Fear scale. Notably, one of its items |aexa was translated in the dictionary as “fiery, ardent,” but qualitative exploration by Thalmayer et al. (2020a) suggested that the term has taken on contemporary meaning of “adept, skilled, exceptional, masterful, in relation to a domain of expertise.” While this new definition was derived from informants throughout the country, they constitute a small sample (*n* = 14). It is possible that more than one meaning was understood for this term, if not for others, among the current study's participants. Using terms with more stable meanings on the KPI might improve measurement properties and lead to more logical and consistent associations.

A measurement issue common to both the KPI and QB6 was revealed by PCA: many respondents appear to have “perseverated,” giving the same response for several subsequent items before changing to a new response. Future analyses should compare this tendency to that in other inventories administered to this and similar samples, where survey research is less familiar than in Western contexts. For a unidimensional inventory such as the SWLS or one with subscales that are expected to correlate and together form a relevant total score, such as the CMHA or DUREL, such a response bias might conceivably improve measurement characteristics rather than attenuate them. This bias would also be nearly invisible where items are administered in a random order, for example in an online survey. The true extent of this tendency may thus be best diagnosed in exactly this context, where items from scales that should not theoretically correlate are presented next each other in a fixed format. Future work could explore this and other response biases (acquiescence, extremeness, moderacy, social desirability, random responding) further. For example, more extensive qualitative piloting could be helpful. Participants could be administered surveys like the KPI and QB6 orally, asking for open-ended responses about how items are perceived and how an answer was arrived at. This might reveal which items are understood as intended and which lead to answers that are basically “guesses” because they are difficult for many participants to relate to their lives. Additionally, qualitative work that asks for free expression in the description of the self and others could make it more obvious how terms are understood and used in context. Strategies that use behavior observations instead of self-report would also be fruitful to explore further.

Along these lines, it is worth considering some particular issues related to the QB6. This inventory was built from items of the International Personality Item Pool (IPIP; https://ipip.ori.org/). This research collaborative was founded in 1992 at the time of the “birth” of the Big Five, and it was based on a goal, to make personality research more open and collaborate across cultures, likely shared by the authors of articles in this special issue. However, like the research base of the Big Five, the international component of the IPIP in practice meant inclusion of German and Dutch items and researchers along with those from North America. Not surprisingly then, IPIP items show themselves to have significant limitations when translated to non-Western contexts; they do not appear to offer particular “international” advantages. They use double negatives, terms such as “rarely” to indicate negation, and colloquialisms, i.e., key terms understood in peculiar ways by contemporary Westerners, all of which are hard to translate effectively. In our case, these items appeared to be hard for participants to consistently understand despite exhaustive efforts to translate them well. An alternative to complex items was tried here, in the form of the single adjectives used on the KPI. It is possible that the lower internal consistencies for QB6 scales could be due to the complexity of the items, and/or due to the concepts being imported and therefore less relevant and understandable to participants. It is not possible to disentangle those effects in this study, but future work should seek to do so.

Five KPI subscales, Temperance, Prosocial Diligence, Gossip, Honesty/Morality, Implacability, inter-correlate with magnitudes of about 0.37 to 0.55 with each other. Humility and Vanity are also a moderately correlated pair. This is not disqualifying for the retention of the 10 KPI subscales: in fact it is the same level at which Big Five Agreeableness, Conscientiousness, and Neuroticism often correlate with each other (e.g., 14 correlation matrices analyzed by Digman, 1997), which is discussed further below, in terms of its facilitation of useful higher order structures, and how these might provide an integrative framework for highly diverse local inventories. At this early stage of scale development, however, it could also be a topic worthy of future work. A limitation of this project to develop the KPI is the small pool of items that were used—with only two items on one scale and only three on two other scales, there is limited potential to drop items to improve psychometric properties. In this sense this study might be viewed as a “pilot” effort to assess the potential for a KPI, and a future study might include a much larger pool of items, and perhaps also short phrases that can more precisely represent the combinations of content in the factor.

As noted, however, orthogonal factors may not be a reasonable standard for a personality inventory, and it is not one that the Big Five meets. The regular pattern of correlations among the Big Five factors has led to their association in two-factor models (e.g., Digman, 1997; DeYoung, 2006). A higher-order structure of the KPI might be similarly logical. Moderate support in the Khoekhoe lexical study (Thalmayer et al., 2020a) for the Big Two (Saucier et al., 2014) and a Pan-Cultural Three model (De Raad et al., 2014) suggest the potential for a higher order structure of the KPI to converge with that of the Big Five/Six, and/or that of lexical studies in many languages. We made a *post hoc* assessment to compare factor scores for a two-factor model of KPI items to a “Big Two” derived from the QB6 scales. Agreeableness, Honesty, and Conscientiousness were combined to estimate Social Self-Regulation, which correlated *r* = 0.52 with the first rotated factor of KPI items; Extraversion and Originality/Openness were combined to estimate Dynamism, which correlated *r* = 0.38 with the second rotated factor of KPI items, indicating some potential.

Future work should assess the relation between the KPI and higher-order models using a more appropriate measure of the Big Two, and should additionally explore its association with cross-cultural three-factor models. Additional KPI items might be added to improve this capability for integrating results. Despite the loss of predictive capability when going to fewer factors, the potential for comparing results with such an approach is important. A disincentive to using indigenous personality inventories is the difficulty of comparing results across contexts. Their integration into simpler, higher-order structures that can be compared at a broad level would provide a key to cross cultural personality research, and a practical alternative to simply exporting the Big Five. This might help personality psychologists to more warmly welcome the rich diversity that naturally arises among contexts, and which illustrates the fascinating ways that human beings have adapted to varied contexts.

For almost all criteria, the group of KPI scales explained more variance than the group of QB6 scales. This is natural given its larger number of items (38 instead of 30). The KPI also had more variables, 10 instead of six. Does this make for an unfair comparison? It has been established that narrower facets have better predictive power (e.g., Mõttus et al., 2017), and we believe that more specific subscales are an advantages of the KPI over the Big Five/Six traits. We believe, however, that this advantage for specificity and more subscales would only temper the results to the extent that more items are used in order to have narrower scales. For predictive efficiency, what's important is the number of items and not the number of variables into which those items are aggregated (illustrated, for example, in Saucier et al., 2020). Including more variables that are relatively independent of one another is an established way to improve prediction; this is an important argument in favor of higher-dimensionality models of personality attributes beyond five or six traits (Saucier and Iurino, 2020).

Hypotheses for specific associations for the KPI, based on face validity and tested in terms of significant regression coefficients, were less likely to be met than those for the QB6, which were based on prior literature. This is unsurprising given that the Big Five and the Big Six have been explored and honed in hundreds if not thousands of studies; personality psychologists know their contours well. The KPI is based on the results of a single prior study and as such is experimental and not yet well-defined. Thus, the tests of associations for the KPI were exploratory and they serve now to better inform us better what these scales capture.

We see, for example, that higher scores on the four items of Resiliency vs. Agitation impressively predicted lower scores on all 14 physical and mental problem scales, in addition to predicting higher income and higher overall religious engagement. Given the significant mental health needs in Namibia (e.g., Feinstein, 2002; Haidula et al., 2003; Shifiona et al., 2006; Bartholomew, 2016), with a suicide rate in the top quartile globally (https://apps.who.int/gho/data/node.main.MHSUICIDE), this scale might be explored as a simple, non-intrusive screener for distress and disorders.

Other scales with strong predictive ability for the criteria used in this study include Temperance, which contrasts religious engagement with substance use and abuse. This scale reflects the significance of religious engagement in Namibia, and the tendency of religious leaders to strongly discourage drinking. As anticipated, higher scores on this scale predicted higher scores on all aspects of religious engagement and fewer problems with substance abuse, also predicting fewer overall psychological disorders, and sleep quality. Temperance additionally had a negative association with income. Based on recent qualitative work, we suspect that this is due to the high cost of alcohol in Namibia relative to wages (and high unemployment). While many people avoid drinking due to their personal and/or religious values, a lack of disposable income is also a reason, especially for young people who might otherwise be interested in trying moderate or social drinking.

Another scale with many associations was Honesty/Morality. Higher scores predicted fewer problems on seven psychological disorder scales, including Work Engagement, Substance Abuse, Anger, both conflict scales, and Psychosis, as well as the overall score. Honesty/Morality scores did not predict religiosity, as was initially expected. The scale's items mostly capture the low end of this trait, referring to being cunning, wicked, and roguish, dishonest, and crooked, and to tormenting others. Indeed this “dishonest-illegal” aspect of moral issues has been shown to associate more with legal codes in the World Values Survey, while “personal-sexual” moral issues are more associated with religions attitudes (Vauclair and Fischer, 2011). This dimension was given the name “Immorality” in the lexical study, but its name was updated here for the KPI to better reflect its content. Interestingly, Temper only associated with sleep problems, not with Anger, although its items focus on anger, temper, aggression and insolence, and its internal consistency was reasonably high for a four-item scale (0.67). Another scale that might benefit from further examination is Vanity, which predicted less rather than more conflict.

Hypotheses for significant regression coefficients for the QB6 based on prior literature are interpreted differently. Where hypotheses are met, they indicate two things: that these scales are valid in Namibia, as they function well enough to pick up expected and appropriate associations; and that these associations between the Big Five and Six traits hold true across cultural contexts. Where the hypotheses are not supported, however, it is not possible to distinguish between these interpretations, and future work will be needed to disentangle them. In many cases they were indeed met, especially for Resiliency, which performed largely as expected, and for Conscientiousness and Honesty, where all hypotheses were met, in addition to many additional, logical associations. Agreeableness and Extraversion, on the other hand, underperformed, mostly failing to associate with scales that they logically should have. Instead of predicting scores on Anger or Conflict scales, higher Agreeableness predicted more Stress. These constructs may have important differences in less individualistic, Western contexts (described further in Thalmayer et al., 2020a). Openness/Originality should be further explored in future work—this dimension was not expected to relate to health-related criteria in the current study, and this it was largely untested here. Of the Big Five and Big Six traits, this may be the least translatable outside the West (e.g., Cheung et al., 2001; Rossier et al., 2017; Thalmayer et al., 2020a).

An important question that this project did not address directly was the extent of the local need for an instrument like the KPI. Our goals in creating this inventory were largely scientific, as described above, to address general questions in personality psychology about the universality vs. cultural specificity of models and measures. It was also developed to provide a locally relevant assessment in the context of a large survey study on mental health. For mental health, the practical needs are clear, and the support for and interest in such work from local leaders and psychologists is strong. More general personality assessment may also be of interest—anecdotally, Namibian labor-ministry psychologists note the lack of local assessment measures for any topic, and their reliance on inventories imported from North America (sometimes after being adapted and modified in South Africa). Future work that seeks to improve the measurement properties or incremental validity of the KPI would ideally be driven by community interests, considering the need and concerns of Khoekhoe-speakers in Namibia, and any values and uses identified by community members and local psychologists.

## Data Availability Statement

A preregistered analysis plan and the data used in this study are available on the Open Science Framework: https://osf.io/pbka4/.

## Ethics Statement

The studies involving human participants were reviewed and approved by University Research Ethics Commission, University of Namibia. The patients/participants provided their written informed consent to participate in this study.

## Author Contributions

AT, GS, and ES contributed to conception and design of the study. AT and ES handled ethical review and study permitting. AT, GS, and SJ made choices on the development of the KPI. SJ and AT led translation of the other inventories. AT, ES, and SJ trained research assistants and monitored data collection. AT planned the statistical analyses and wrote the first draft of the manuscript. AT performed all analysis, with GS contributing to interpreting results. All authors contributed to manuscript revision, read, and approved the submitted version.

## Conflict of Interest

The authors declare that the research was conducted in the absence of any commercial or financial relationships that could be construed as a potential conflict of interest.
